# Beneficial effect and mechanism of walnut oligopeptide on *Lactobacillus plantarum* Z7

**DOI:** 10.1002/fsn3.2029

**Published:** 2021-01-08

**Authors:** Ting Ding, Yong Li

**Affiliations:** ^1^ Department of Nutrition and Food Hygiene School of Public Health Peking University Beijing China

**Keywords:** biofilm, EPS, *Lactobacillus plantarum* Z7, quorum sensing, WOPs

## Abstract

Prebiotics can stimulate the growth and activity of probiotics and have a variety of physiological functions. However, the study of walnut oligopeptides as prebiotics to promote probiotics is rarely reported. Therefore, in order to explore the beneficial effect of walnut oligopeptide (WOPs) on *Lactobacillus plantarum* Z7, WOPs was added to the medium of *L. plantarum* Z7, and the utilization of WOPs, the effect of WOPs on the biofilm, extracellular polymeric substances, and bacterial death were explored. The results showed that the growth‐promoting effect was strengthened with the increasing concentrations of WOPs. The content of bacterial biofilm and EPS increased significantly, and the number of dead bacteria decreased. The beneficial effect of WOPs was probably because that it enhanced the secretion of biofilm which was regulated by bacterial quorum sensing system and promoted the ability of bacteria to resist the adverse environment, thus promoting the growth and reproduction of bacteria.

## Graphical abstract

Walnut oligopeptide (WOPs) promoted the growth and reproduction of bacteria and increased the secretion of bacterial biofilm and EPS, which make WOPs to be a new and potential prebiotic to promote the growth and quality nutritional value of related products.

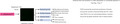

## 1 INTRODUCTION

Probiotics are a kind of bacteria that are colonized in human intestinal tract and reproductive system, which can improve the micro ecological balance and play a beneficial role in human body (Sanders et al., 2013). It is not only a kind of food supplement, but also has been gained more and more attention to its nutrition, health care, and therapeutic effect. In recent years, probiotics have been found to play an important role in the treatment of dermatitis, asthma, gout, diabetes, hepatogenic encephalopathy, gastrointestinal infection, and other diseases (Campaniello et al., 2015; Cordina et al., 2011; Delley et al., 2015; Reid, 2015). However, the number of viable bacteria decreased rapidly in the process of processing, transportation, storage, sales, and consumption. External factors such as temperature, substrate, and oxygen can affect the activity of the strains. Some probiotics have high requirements for nutritional conditions and poor resistance to low pH environment. They are extremely sensitive to oxygen and are difficult to maintain their activity (Doleyres & Lacroix, 2005). In addition, probiotics need to pass through the stomach environment after eating, but a large number of active probiotics died due to the bactericidal effect of gastric acid. This also reduces the number of viable bacteria in many commercial probiotic products to invalid amount, which can not achieve the beneficial effect on human body. It is an effective way to improve the survival rate of probiotics by adding growth‐promoting factor (prebiotic) to probiotics. The prebiotics widely used now include inulin, fructooligosaccharide, galactooligosaccharide, and dextran, but these fast‐fermenting oligosaccharides have many disadvantages, which can cause human diarrhea and flatulence (Puupponen‐Pimiä et al., 2002). Therefore, it is of great significance to find and explore the substances and methods that have beneficial effects on probiotics and improve their resistance to high temperature, oxygen, gastric acid, bile, and other adverse environmental factors, which will enhance the maintenance of human physiological health.

*Lactobacillus plantarum* belongs to probiotic family, which has many functions, such as regulating intestinal flora, regulating blood lipid, enhancing the anti‐oxidation ability of the body, participating in the immune response, and reducing cholesterol level. It has also been widely used in the food industry (Behera et al., 2018). Previous studies have shown that the addition of bioactive peptides to probiotics can affect the fermentation process and promote the growth and reproduction of microorganisms (Cudennec & Ravallec, 2013; Yu et al., 2017). Bioactive peptide is a compound composed of two or more amino acid molecules linked by peptide bond, which can participate in physiological activities and play an important physiological role in human body. Some peptides have physical and chemical properties (good cellular diffusion and permeability, small size, low toxicity/side‐effects, etc.) and special physiological activities that some proteins do not have, such as promoting fermentation, lowering blood pressure, and anti‐oxidation. Thus, bioactive peptides have obtained a great degree of attention and interest. Compared with the same concentration of amino acids, it is easier to be absorbed by intestinal tract. Walnut oligopeptide (WOP) is a kind of bioactive peptide, which has many bioactivities, such as lipid‐lowering, anti‐oxidation, and antifatigue (Robbins et al., 2012), but its beneficial effect on probiotics has been rarely studied. Therefore, the beneficial effect and mechanism of walnut oligopeptide on probiotics *L. plantarum* Z7 were studied to provide reference for improving the survival rate of probiotics and the stability of commercial products.

## 2 MATERIALS AND METHODS

*Lactobacillus plantarum* Z7 was stored in our laboratory. The strain (1%) was cultured in the sterilized MRS broth medium for 24 hr (37°C, 160 rpm). MRS broth medium was purchased from Beijing Aoboxing Biotechnology Co., Ltd. Walnut oligopeptide is a light yellow solid powder, mainly composed of small oligopeptide with molecular weight less than 1,000, which contains more glutamic acid, aspartic acid, arginine, and leucine. It was provided by Beijing Tianpeptide Biotechnology Co., Ltd.

### 2.1 Functional properties of WOPs on the growth of *Lactobacillus plantarum* Z7

The MRS liquid culture medium was divided into 20‐ml anaerobic tubes and sterilized for 15 min (121°C). Then, 0.5%, 1.0%, 1.5%, 2.0% WOPs, and 1% *L. plantarum* were added into the MRS liquid culture medium, respectively. MRS medium was taken as blank control, and fructooligosaccharide (2.0%) was taken as control. The tubes were cultured at 37°C for 24 hr (160 rpm). Samples were taken every hour to determinate the changes in the number of bacteria. The determination was carried out by using double broth dilution method. Three appropriate dilution were added to MRS solid medium (1.5% agar was added into MRS liquid medium) and mixed. Then, the plates were incubated at 37°C for 48 hr to count the number of colonies and drew the bacterial growth curve. Every dilution was repeated for 3 times.

### 2.2 Exploration of the growth of *Lactobacillus plantarum* Z7 by microbial growth dynamics model

The growth kinetics model can be used to calculate the lag phase and maximum specific growth rate of microorganisms. Commonly used models of microbial growth dynamics include Gompertz model, Modified Gompertz model, Modified logistic model, Monod model, Baranyi and Roberts model, Richards model, Stannard model, and Schnute model (Zwietering et al., 1990). The Modified Gompertz model and Modified Logistic model were used to describe the change rule of *L. plantarum* Z7 with different concentrations of walnut oligopeptides by nonlinear fitting.

1. Modified Gompertz model:

The equation of Modified Gompertz model is:

$$\log\left(N_{t}\right)=\log\left(N_{0}\right)+\log\left(\frac{N_{\max}}{N_{0}}\right)\times \exp\left\{‐\exp\left[\frac{\mu_{\max}\times 2.718}{\log\left(N_{\max}/N_{0}\right)}\times \left(\lambda ‐t\right)+1\right]\right\}$$

In the equation, *t* is the time (hr); *N*(t) is the number of bacteria at time *t*; *N*_max_ and *N*_0_ are the maximum and initial number of bacteria, respectively (cfu/ml); *μ*_max_ is the maximum specific growth rate (h^−1^); and *λ* is the growth lag phase (h).

- Modified Logistic model

The equation of Modified Logistic model is:

$$\log\left(N_{t}\right)=\log\left(N_{0}\right)+\frac{A}{1+\exp\left(4*\mu_{\max}\frac{\lambda ‐t}{A}+2\right)}$$

In the equation, *t* is the time (hr); *N*(*t*) is the number of bacteria at time *t* (cfu/ml); *N*_0_ is the initial number of bacteria (cfu/ml); A is the fitting parameter; *μ*_max_ is the maximum specific growth rate (h^−1^); and *λ* is the lag phase (h).

### 2.3 Utilization of WOPs by *Lactobacillus plantarum* Z7

Fluorescein isothiocyanate (8.6 mg) was dissolved in 0.2 ml of 0.1 M KOH solution and mixed. Then, 1.6 ml of 0.1 M carbonate buffer (Na_2_CO_3_/NaHCO_3_) with pH of 8.3 was added. WOPs solution (0.5%, 1 ml) was added to the above solution and mixed. The WOPs was placed in 4°C for 4 hr under dark conditions to be labeled by fluorescein isothiocyanate. The 0.5% labeled peptide solution and 1% *L. plantarum* Z7 were added to MRS medium. After cultured at 37°C for 24 hr (160 rpm), 2.5 μl of bacterial fluid was added in a clean slide glass substrates, and then, it was observed with the laser scanning microscope (CLSM; TCS‐SP8; Leica Company). Maximum emission wavelength: 520–530 nm, maximum absorption wavelength: 490–495 nm.

### 2.4 Effect of WOPs on biofilm formation of *Lactobacillus plantarum* Z7

#### 2.4.1 Quantitative determination of biofilm

*Lactobacillus plantarum* Z7 (1%) was cultured in MRS broth medium for 24 hr (37°C, 160 rpm). Then, 20 μl bacterial culture and 100 μl MRS broth medium were added to 96 well plate, and 0.5%, 1.0%, 1.5%, and 2.0% WOPs were added to the well, respectively. Fructooligosaccharide with 20 μl bacterial culture was used as control, and MRS broth with 20 μl bacterial culture was used as blank control. The 96‐well plates were placed in 37°C incubator for 48 hr. After culture, the wells were washed with sterile PBS (7 mM Na_2_HPO_4_, 3 mM Na_2_H_2_PO_4_, 130 mM NaCl, pH 7.4) buffer for three times to remove the free bacteria. XTT was prepared as 0.5 mg/ml stock solution with PBS and filtered with 0.22 μm filter membrane and stored at −70°C. Vitamin K3 was dissolved in acetone (10 mM). Vitamin K3 was added to XTT to reach the concentration of 1 μM. Then, 200 μl XTT‐vitamin K3 solution was added to 96‐well plate and the plate was placed at 37°C for 2 hr under dark conditions and then tested at 490 nm with microplate reader. Each sample was repeated four times to take the average value.

#### 2.4.2 Microscopic observation of biofilm

*Lactobacillus plantarum* Z7 (1%) was cultured in MRS broth medium for 24 hr (37°C, 160 rpm). Then, 200 μl bacterial culture, 20 ml MRS broth medium, and 2.0% WOPs were added to sterile petri dishes. MRS broth with 200 μl bacterial culture was used as control. Sterile cover glass was put into the dishes and cultured at 37°C for 48 hr. After culture, the cover glass was washed for 3 times with ultrapure water and dyed with 0.4% crystal violet for 20 min. The bacterial biofilm was observed under oil microscope.

#### 2.4.3 Observation of biofilm by CLSM

*Lactobacillus plantarum* Z7 (1%) was cultured in MRS broth medium for 24 hr (37°C, 160 rpm). Then, 200 μl bacterial culture, 20 ml MRS broth medium, and 2.0% WOPs were added to sterile petri dishes. MRS broth with 200 μl bacterial culture was used as control. A polished zinc (0.5 mm × 0.5 mm × 0.3 mm) was placed at the bottom of each plate to make the biofilm adhere to the zinc. The plates were placed in a 37°C incubator for 48 hr. After culture, the zinc was took out and washed with sterile water for three times to remove free cells. Then, it was fixed with 2.5% glutaraldehyde for 30 min, washed, and dried for 30 min. The biofilm was dyed by 0.01% acridine orange (w/v, dissolved with PBS) solution in dark condition for 15 min. Then, it was washed for three times with sterile PBS buffer and dried for 30 min. Approximately 10 μl of sealing agent was added, and it was observed with CLSM. Observation conditions: emission wavelength: 525 nm, excitation wavelength: 488 nm.

### 2.5 Effect of WOPs on EPS of *Lactobacillus plantarum* Z7

#### 2.5.1 Scanning electron microscope observation of EPS

The zinc as described above was took out and washed slowly with sterile water for three times to remove the free bacteria. Then, it was dried for 30 min and immersed in 2%–5% glutaraldehyde (*v*/*v*) for 4 hr. Then, the zinc was immersed in 50%, 70%, 80%, and 90% (*v*/*v*) ethanol for 10 min and 100% ethanol for twice (15 min each), and finally, it was immersed in 25% isoamyl hexanoate for twice (15 min each). After drying, the zinc was treated by spraying gold and tested by scanning electron microscope (SEM).

#### 2.5.2 Determination of the chemical composition of EPS

The change of chemical composition of EPS was measured by Raman spectroscopy (HORIBA Scientific Company). The zinc as described above was washed with PBS buffer to remove the free bacteria and dried for 30 min. Then, it was tested by Raman spectrum. Laser source wavelength: 532 nm; laser source power: 25 mW; 50 times objective lens; 5 times of exposure; detection spectrum range: 100–3,500 cm^−1^.

### 2.6 Effect of WOPs on bacterial death of *Lactobacillus plantarum* Z7

*Lactobacillus plantarum* Z7 (1%) was cultured in MRS broth medium for 24 hr (37°C, 160 rpm). Then, 100 µl bacterial culture, 10 ml MRS broth medium, and 2.0% WOPs were added to 20 ml anaerobic tubes. Moreover, 10 ml MRS broth with 100 µl bacterial culture was used as control. The tubes were cultured for 24 hr (37°C, 160 rpm), and then, the bacterial culture was washed with normal saline and centrifuged for 5 min (2146.56 g). Then, it was fixed by 70% alcohol overnight at 4°C and washed twice with PBS. Subsequently, it was stained with PI staining kit at 4°C for 30 min, and the unstained *L. plantarum* Z7 was used as negative control. The bacteria was filtered with 300 mesh nylon mesh and detected by flow cytometry (Cytoflex; Beckman Company).

### 2.7 Statistical analysis

The results are expressed as the mean ± standard deviation (*SD*) and were analyzed through one‐way ANOVA by SPSS Statistics 16.0 (SPSS, Inc.) Significant differences are displayed at *p* < .05. All tests were repeated three times.

## 3 RESULTS

### 3.1 Effect of WOPs on the growth of *Lactobacillus plantarum* Z7

**FIGURE 1:**
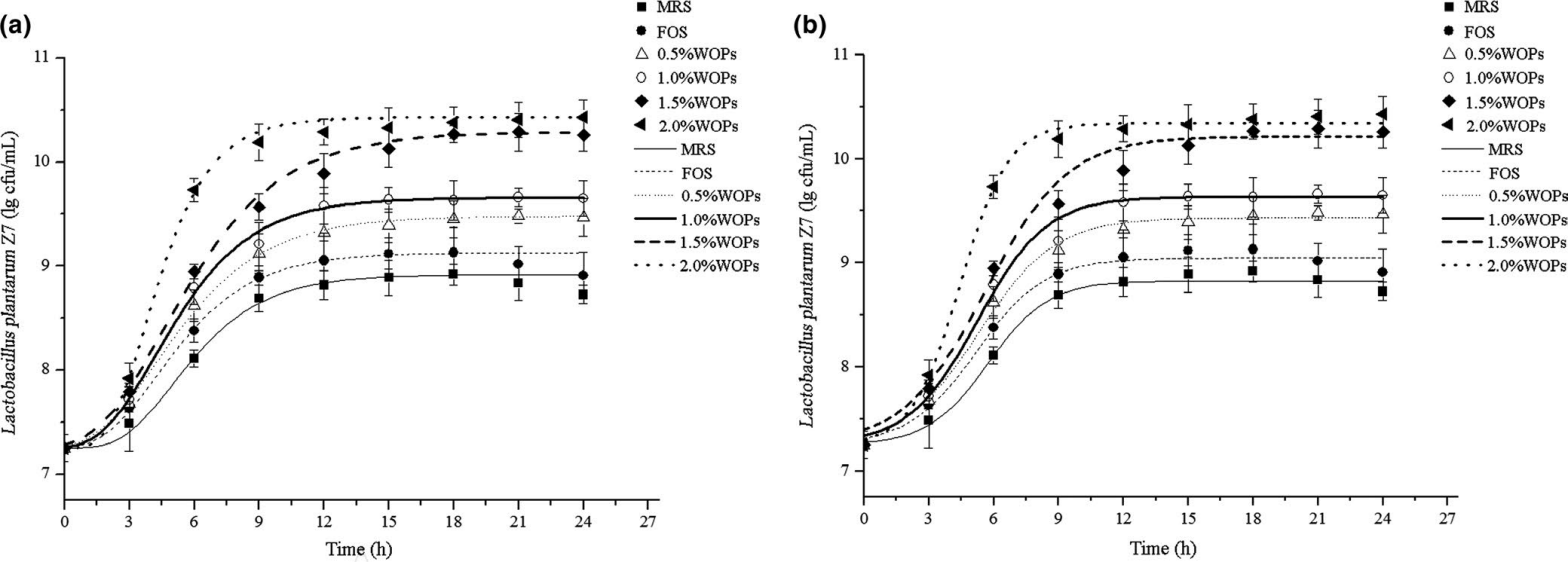
Growth curve of *Lactobacillus plantarum* Z7. (a) Modified Gompertz equation; (b) Modified Logistic equation

The effect of WOPs on the growth of *L. plantarum* Z7 was studied by determination of its growth curve. The change of the number of *L. plantarum* Z7 with time was showed in Figure 1. It could be seen from the figure that *L. plantarum* Z7 proliferated rapidly in MRS medium, and the bacteria entered logarithmic growth period after 3 hr with short lag phase. The curve tended to be stable, and the bacteria entered stable period after 12 hr. After 18 hr, the pH value of fermentation liquid was reduced, and the growth environment of bacteria got worse gradually, which led to the slow metabolism of bacteria and the decline of the number of living bacteria due to the continuous accumulation of lactic acid and other metabolites. Then, the bacteria growth curve entered the decline period. The number of bacteria increased significantly after the fructooligosaccharide (FOS) was added to the bacterial culture medium when compared to the control group. But after 18 hr, the bacteria also entered the decline period. However, the stimulating effect of the culture medium with WOPs was better than that of FOS. The number of living bacteria in the experimental group was significantly higher than that of control group, and the higher the concentration of WOPs, the better the stimulating effect. The logarithmic period of bacterial growth was advanced, and the stable period was also prolonged. During the stable period, the number of living bacteria in the medium supplemented with 0.5%, 1.0%, 1.5%, and 2.0% WOPs reached 3.02 × 10^9^, 4.57 × 10^9^, 1.95 × 10^10^, and 2.69 × 10^10^ cfu/ml, respectively. The results showed that WOPs could enhance the metabolic rate of bacteria and promote the proliferation of probiotics.

The growth and change of *L. plantarum* Z7 were studied by using the model of microbial growth kinetics, and the growth curve of bacteria was fitted by Modified Gompertz equation and Modified Logistic equation, respectively. It was found that the growth curve of bacteria could be well fitted by the Modified Gompertz equation and Modified Logistic equation (Figure 1). Most of the data points were on or close to the fitting curve. The change and growth dynamics of *L. plantarum* Z7 with time could be well reflected by the two equations. The fitting effect of different microbial growth kinetics equation could also be determined by the coefficient of determination (*R* ^2^). The larger the *R* ^2^ value, the better the fitting degree between microbial growth curve and microbial growth kinetics equation. It could be seen from Table S1 that the *R* ^2^ values were different when the growth data of *L. plantarum* Z7 were fitted by different microbial growth kinetics equations. The maximum specific growth rate (*μ*_max_) and lag phase (*λ*) of the bacterial growth curve could be obtained by the Modified Gompertz equation. It was found that *R* ^2^ > .967 and *R* ^2^ > .982 when the bacterial growth curve was fitted by Modified Gompertz equation and Modified Logistic equation, respectively, which also indicated that the two equations could well describe the growth dynamics of microorganisms.

Growth rate is an important parameter to study the growth rule of microbial population under the condition of logarithmic growth. It can not only estimate the bacterial concentration at a certain time, but also be an indicator to judge the relationship between microbial growth and environment. The nonlinear fitting parameters of microbial growth kinetics equation are shown in Table S1. It could be seen from the table that the addition of WOPs and FOS had a significant effect on the maximum specific growth rate and lag phase of bacteria. The maximum specific growth rate (*μ*_max_) increased, and the lag phase (*λ*) shortened when the bacterial culture medium was supplement with WOPs and FOS. The effect of WOPs on *μ*_max_ and *λ* was more obvious than that of FOS. It could be concluded from the above results that WOPs could promote bacterial proliferation and make them enter the logarithmic growth period ahead of time. WOPs can be used as a growth‐promoting factor of probiotics, which will have great application value.

### 3.2 Utilization of WOPs by *Lactobacillus plantarum* Z7

**FIGURE 2:**
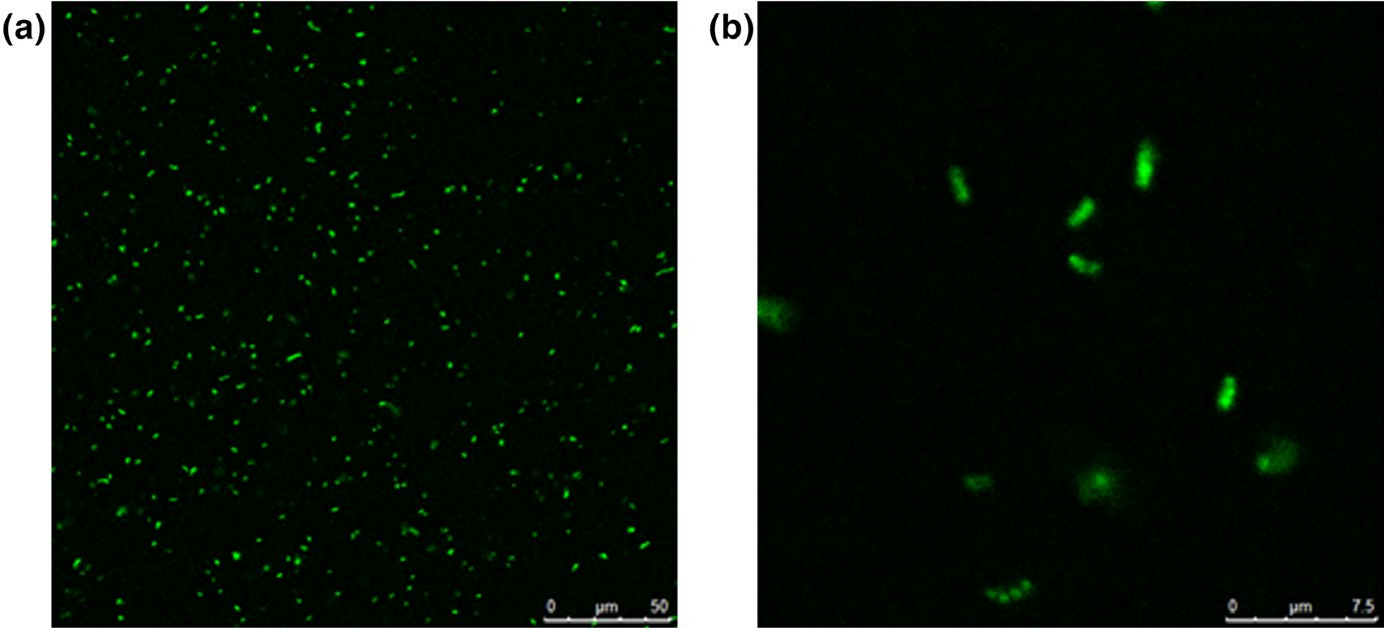
Utilization of walnut oligopeptides (WOPs) by *Lactobacillus plantarum* Z7 under CLSM

The WOPs could be stained by fluorescein isothiocyanate in the culture medium of *L. plantarum* Z7. The isothiocyanate group in fluorescein isothiocyanate could covalently combine with amino acids in the peptide, so the utilization of WOPs by bacteria could be observed under confocal laser microscope. CLSM test found that *L. plantarum* Z7 could make good use of WOPs (Figure 2a). The fluorescence labeled WOPs were found in the cytoplasm of most of the bacteria in the enlarged picture (Figure 2b), indicating that the WOPs could freely enter the bacteria through the cell membrane to provide energy for the bacteria to carry out various metabolic activities.

### 3.3 Effect of WOPs on biofilm of *Lactobacillus plantarum* Z7

**FIGURE 3:**
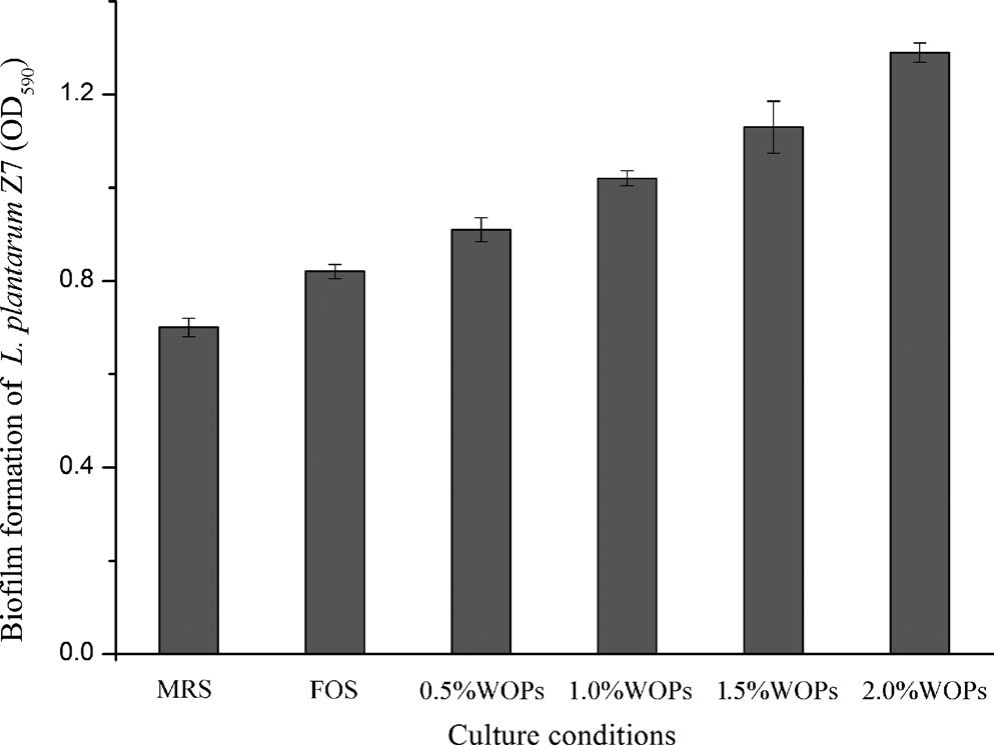
Quantitative determination of the effect of walnut oligopeptides (WOPs) on the biofilm of *Lactobacillus plantarum* Z7

It could be seen from the quantitative determination of WOPs on the biofilm of *L. plantarum* Z7 in Figure 3 that both FOS and WOPs could promote the production of bacterial biofilm, but the promotion of WOPs was more significant than that of FOS. Moreover, the higher the concentration of WOPs, the more significant the promotion effect. The content of bacterial biofilm increased by 30.0%, 45.71%, 61.43%, and 84.29%, respectively, when the bacterial culture medium was added with 0.5%, 1.0%, 1.5%, and 2.0% concentrations of WOPs. The quantitative results showed that WOPs significantly promoted the production of biofilm of *L. plantarum* Z7.

**FIGURE 4:**
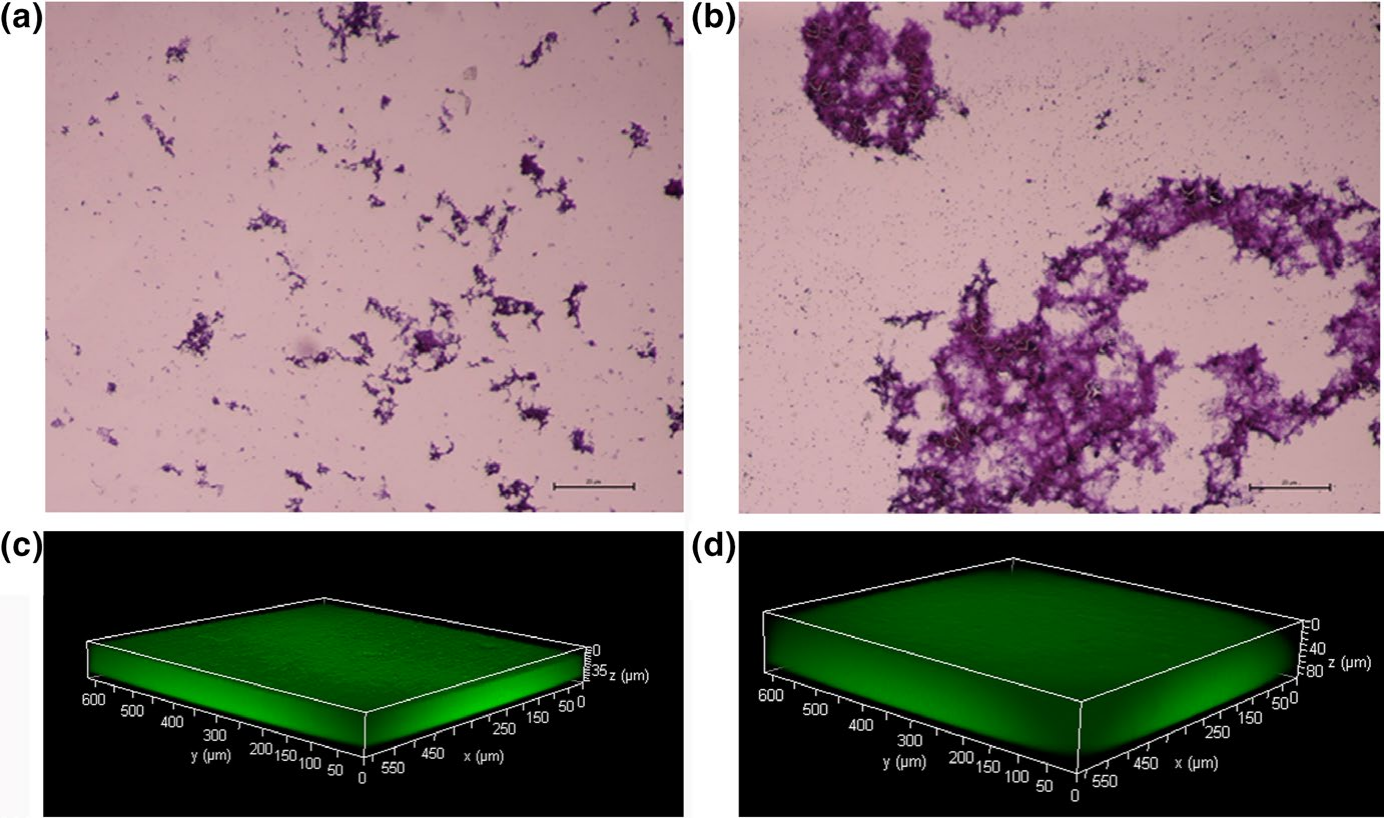
The effect of walnut oligopeptides (WOPs) on the biofilm of *Lactobacillus plantarum* Z7 by optical microscope (a, b) and CLSM (c, d); (a, c) Control; (b, d) Addition of 2.0% WOPs

It could be seen from Figure 4 that the biofilm formed by bacteria was dyed by crystal violet (Figure 4a,b). The bacterial distribution of *L. plantarum* Z7 was relatively scattered when the culture medium was not supplemented with WOPs. However, the cells gathered into groups and formed a large and dense biofilm when the WOPs was added. Moreover, the bacteria wrapped in the biofilm and formed a complex three‐dimensional structure. The thickness of biofilm was observed by CLSM (Figure 4c,d). It was found that the thickness of biofilm produced by bacteria without WOPs was relatively thin, with an average thickness of about 35 μm, while the thickness of biofilm produced by bacteria with WOPs increased significantly, with an average thickness of about 80 μm. These results indicated that the WOPs significantly stimulated the production of biofilm of *L. plantarum* Z7. The results of optical microscope were consistent with that of CLSM.

### 3.4 Effect of WOPs on EPS of *Lactobacillus plantarum* Z7

**FIGURE 5:**
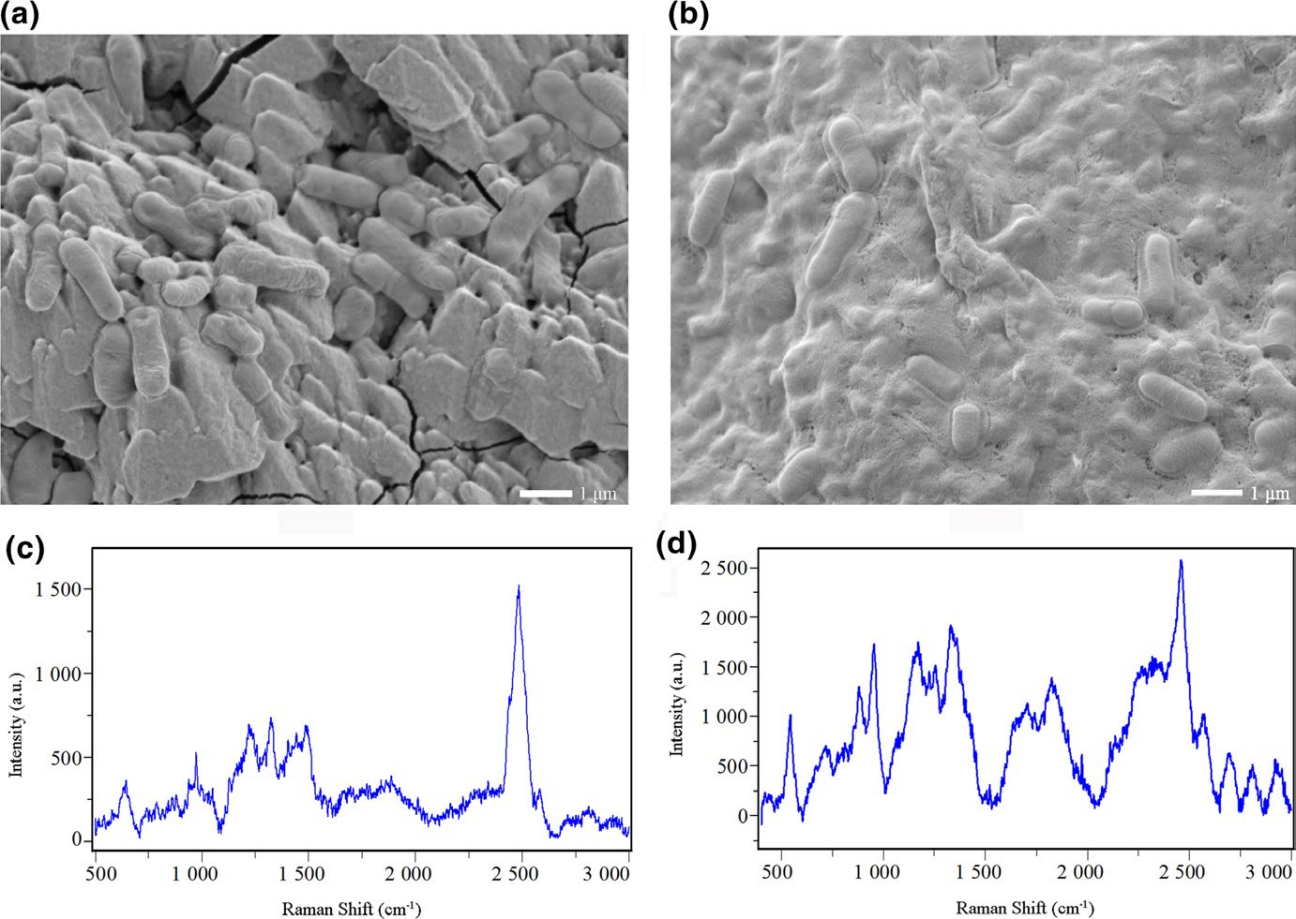
Effect of walnut oligopeptides (WOPs) on EPS of *Lactobacillus plantarum* Z7 observed by Scanning electron microscope (SEM) (a, b) and Raman spectroscopy (c, d); (a, c) Control; (b, d) Addition of 2.0% WOPs

The effect of WOPs on EPS production of *L. plantarum* Z7 was observed by SEM. It was found that EPS produced by bacteria was little when the culture medium was not supplemented with WOPs (Figure 5a). However, it produced a large number of dense EPS, and the bacteria wrapped in EPS, forming complex biofilms when WOPs were added (Figure 5b). The effect of the addition of WOPs on the chemical composition of EPS of *L. plantarum* Z7 could be detected by Raman spectroscopy. Figure 5c,d showed that the peak intensity of Raman spectrum of EPS produced by bacteria increased after WOPs were added. The corresponding substances of the Raman spectrum characteristic peak of EPS of bacteria were shown in Table S2.

By comparing the corresponding substances of the characteristic peaks of Raman spectrum, it could be seen that the main components of the EPS produced by *L. plantarum* Z7 were protein, carbohydrate, nucleic acid, and lipid. The Raman spectrum showed that the corresponding substances of EPS in control group in the 720–730 cm^−1^ and 810–820 cm^−1^ were mainly adenine, tryptophan, and nucleic acid. These vibrations were mainly caused by CH_2_ and C‐O‐P‐O‐C. Bands identified in the range of 1,001–1,005 cm^−1^ were phenylalanine; and between 1,030 and 1,130 cm^−1^, carbohydrate was identified as the main component, and the deformation vibration was mainly caused by ‐C‐C‐, C‐O, and C‐O‐H. Bands at 1,200–1,290 cm^−1^ were attributed to amide III; characteristic bands at 1,320 cm^−1^ indicated that the corresponding substance was mainly protein, which was mainly caused by C‐H group; bands at 1,650–1,680 cm^−1^ indicated that amide I and unsaturated lipid were the main components; between 1,855–2,920 cm^−1^, CH_2_ group, CH_3_ group, sporopollenin, and lipid were identified in the EPS produced by *L. plantarum* Z7. The Raman spectrum showed that the carbohydrate corresponding to the bands between 544–553 cm^−1^ and 1,030–1,130 cm^−1^ increased significantly after the addition of WOPs. Protein bands in the EPS found at 1,320 cm^−1^ also increased significantly; and the amide I, unsaturated lipid, sporopollenin, lipid, and other substances which were corresponding to the bands of 1,650–1,680 cm^−1^ and 2,920–1,855 cm^−1^ increased significantly. The above results showed that the addition of WOPs significantly stimulated the production of EPS in *L. plantarum* Z7.

### 3.5 Effect of WOPs on bacterial death of *Lactobacillus plantarum* Z7

**FIGURE 6:**
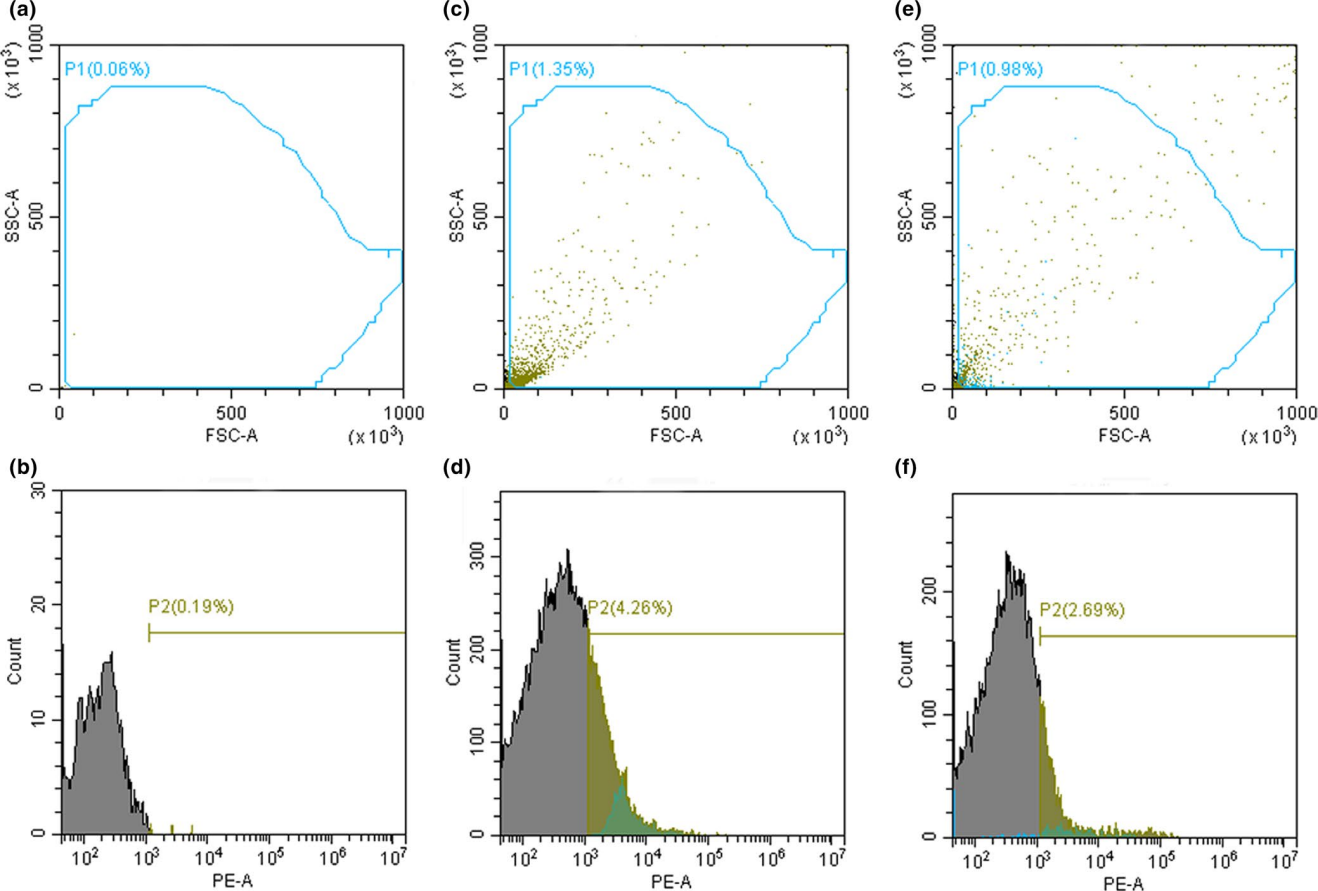
Effect of walnut oligopeptides (WOPs) on bacterial death of *Lactobacillus plantarum* Z7 by flow cytometry. (a, b) Negative control; (c, d) Control; (e, f) Add 2.0% WOPs

Flow cytometry can be used to count bacteria, which can quickly and sensitively determine the death of bacteria. This method needs few samples and is easy to operate, which makes it one of the best methods to detect the dead cells at present. From the scatter diagram analysis (Figure 6a,c,e), the forward scatter (FSC) reflected the size and activity of bacteria. The more the x‐axis to the right, the larger the cell volume; the vertical axis reflected the particle size and structural changes of bacterial cells, and the more upward, the more complex the refractive substances in cells. Because the total events of the scatter plot are easy to make a general statistics of the cell fragments, which leads to the inaccurate bacterial count, however, through the P1 circle gate count, the bacteria with uniform morphology can be counted, and the impact of bacterial fragments and adhesion bacteria can be excluded to the greatest extent; thus, the bacterial count data are more accurate. It could be seen from the scatter diagram that after the addition of WOPs, the volume of bacteria and the internal particle size increased, which may be due to the increase of bacterial biofilm and EPS. After the addition of WOPs, bacteria agglomerated and formed a complex three‐dimensional structure, which made the total volume and internal particle size of bacteria increased. PI is a fluorescent dye that can bind to DNA and can not freely pass through the cell membrane of living cells. When the cell dies, the cell permeability increases. PI can pass through the damaged cell membrane and enter the cell to bind to DNA, and under the excitation laser of 488 nm, red fluorescence can be detected at the wavelength of 660 nm. Therefore, PI can be used to distinguish living cells from dead cells. Through histogram analysis (Figure 6b,d,f), the horizontal axis reflected the fluorescence intensity of bacterial DNA content, and the higher the peak value was, the stronger the fluorescence intensity of cells was. The fluorescence intensity of the cells changed, and the peak value of the figure shifted to the left when the WOPs were added, suggesting that the death rate of bacteria decreased compared to the control. These results indicated that WOPs could significantly enhance the activity of bacteria and reduce the occurrence of bacterial death.

## 4 DISCUSSION

*Lactobacillus plantarum* is an important probiotic. In the process of fermentation, it can produce a lot of lactic acid, acetic acid, extracellular polysaccharide, and other substances. These substances are very important to adjust the balance of intestinal flora, promote intestinal digestion, control the toxins in the body, and enhance the immune function. However, probiotics are often affected by adverse external conditions in the processing and marketing. Moreover, the activity and survival rate of probiotics would significantly reduce when they pass through the stomach environment and enter the human intestinal tract in the form of oral administration (Doleyres & Lacroix, 2005; Hoover, 1993; Ranadheera et al., 2010; Ross et al., 2005; Shah, 2000). Therefore, the proliferation and culture of probiotics have become more and more important. It has been reported that the growth and activity of microorganisms could be stimulated by the addition of exogenous peptide substances. Some enzymatic hydrolysis of proteins from animal and plant sources for the production of bioactive peptides has been taken general attention. Arakawa *et al*. evaluated the demand of peptide for the growth of *Lactobacillus gasseri* and evaluated the efficacy of peptide release by enzymatic proteolysis on growth of *L. gassei* in milk. They found that peptide could be used as a high‐quality milk‐derived nitrogen source to promote the growth of probiotics, and the growth of probiotics required peptide from milk source, rather than free amino acids or proteins (Arakawa et al., 2015). Dave *et al*. found that protein hydrolysate could accelerate the growth and reproduction of probiotics in the fermentation process (Dave & Shah, 1997). Martone *et al*. obtained a highly soluble fish protein hydrolysate from hake filleting waste. They found that the protein hydrolysate could promote the cell growth of Halobacterium salinarum, *Escherichia coli*, *Bacillus subtilis*, and *Staphylococcus epidermidis* and could be used as an alternative substrate to culture microorganisms (Martone et al., 2005). Mccomas and coworkers also found that the whey protein hydrolysate could increase the growth of probiotics (*Bifidobacterium longum* S9, *L. acidophilus* O16, and *L. acidophilus* L‐1) in combination with different yogurt cultures in milk. And the whey protein hydrolysate could be used as prebiotics for the growth of probiotics (Mccomas & Gilliland, 2003). Castro *et al*. reported that protein hydrolysate of soy protein isolate, bovine whey protein, and egg white protein positively stimulated the growth of *Streptococcus thermophilus*, *Lactobacillus delbrueckii*, *Lactobacillus acidophilus*, and *Bifidobacterium lactis* (Castro & Sato, 2014). Zhang *et al*. isolated three novel growth‐stimulating peptides, *that is*, H‐2‐A, F2‐c, and F2‐b from sodium caseinate hydrolysates produced by papain. They found that the peptide could promote the growth of *Streptococcus thermophilus* and *Lactobacillus delbrueckii* subsp. *Bulgari*cus (Zhang et al., 2011).

Walnut can be used as medicine and food with a long history. It has high nutritional value and is rich in unsaturated fatty acids, protein, vitamins, folic acid, polyphenols, and other active ingredients (Sze‐Tao & Sathe, 2000). WOP is a kind of bioactive peptide, which is obtained from walnut by enzymolysis. It was found that WOPs showed a variety of physiological activities (Li et al., 2017, 2018; Liao et al., 2016), but there was little research on its beneficial effect on probiotics. Therefore, it is feasible to explore the stimulation effect of WOPs on the growth of probiotics and its long‐term effect as a new prebiotic, which will have great application potential.

When WOPs were added to the culture medium of *L. plantarum* Z7, the growth rate of bacteria was accelerated, the logarithmic period was advanced, the stable period was prolonged, and the death rate was decreased. These results indicated that the WOPs enhanced the metabolism rate of bacteria, which may be due to that the WOPs improved the tolerance of bacteria to adverse environment. Similarly, Madureira and coworkers also found that probiotic whey cheese promoted the tolerance of *Lactobacillus casei*, *Lactobacillus acidophilus*, and *Bifidobacterium animalis* to simulated digestive tract (Madureira et al., 2011). Extracellular polymer is a kind of polymer secreted by bacteria in certain environment. Bacteria are wrapped in these polymers to form complex biofilm structures, through which bacteria can resist the attack of fungicides and other toxic substances (Danese et al., 2000; Hall‐Stoodley et al., 2004). The production of biofilm and EPS is regulated by quorum sensing (QS) system (Liu et al., 2007; Martins et al., 2014). QS is a communication mechanism between bacteria, and it is also a process in which bacteria adjust their physiological and biochemical characteristics according to their population density. Bacteria can produce, release, and recognize extracellular signal molecules called autoinductors (AIs). AIs will accumulate with the increase of bacterial density, and once the threshold concentration of population density is reached, the signal molecules will be sensed by a variety of receptors and then activate or inhibit the expression of specific target genes, leading to a variety of bacterial colony behaviors, such as biofilm production, extracellular enzyme secretion, plasmid transfer, and antibiotic synthesis (Ahumedo et al., 2010; Kim et al., 2015; Wang et al., 2015). It has been proved that probiotics in biofilm state have more significant immunomodulatory effect than probiotics in planktonic state (Rieu et al., 2014). Cheow *et al*. found that probiotics in high‐density biofilm state have better freeze‐drying resistance, heat resistance, and acid resistance compared with conventional probiotics (Cheow et al., 2014). Chew and coworkers found that the probiotics *Lactobacillus rhamnosus* GR‐1 and *Lactobacillus reueri* RC‐14 had the ability to inhibit or interfere with the formation of the biofilm of the pathogenic bacteria *Candida glabra* (Chew et al., 2015). Vuotto *et al*. found that probiotics could be used to fight diseases related to biofilm infectious (Vuotto et al., 2014). Therefore, the biofilm of *L. plantarum* Z7 thickened and EPS secretion increased after the addition of WOPs. The beneficial effect of WOPs was probably due to that it promoted the production of biofilm and EPS which was regulated by QS system, thus increased bacterial resistance to adverse environment. The formation of probiotic biofilm is affected by many factors. It has been reported that the lack of nutrients in the culture medium and the restriction of the supply of carbon source nutrients would promote the formation of biofilm of *Lactobacillus rhamnosus* GG (Lebeer et al., 2007). Slizova *et al*. found that the content and types of Tween‐80 and sugar in the culture medium played an important role in the formation of biofilm of *Lactobacillus reuteri* (Slizova et al., 2015). Therefore, the addition of WOPs can be used to improve the formation of biofilm of *L. plantarum* Z7, which has guiding significance for improving the activity of probiotics.

## 5 CONCLUSION

This study investigated the effects of WOPs on the growth curve, biofilm formation, EPS production, and death rate of *L. plantarum* Z7. It was found that WOPs not only promoted the growth and reproduction of bacteria, but also reduced the number of dead cells. The secretion of bacterial biofilm and EPS increased significantly after the addition of WOPs, so the promoting effect of WOPs on the growth of *L. plantarum* Z7 was probably related to the quorum sensing system of bacteria. WOPs can be used as a new and potential prebiotic to promote the growth and quality nutritional value of related products.

## CONFLICT OF INTEREST

All authors declare no conflict of interest.

## ETHICAL APPROVAL

This study does not involve any human or animal testing.

## INFORMED CONSENT

Written informed consent was obtained from all study participants.

## Supporting information

Table S1

Table S2
