# Supplementary material for: Beneficial effect and mechanism of walnut oligopeptide on Lactobacillus plantarum Z7
Source: Food Sci Nutr. 2021 Jan 8;9(2):672–81. doi: 10.1002/fsn3.2029 (PMC7866578; doi:10.1002/fsn3.2029)
Supplement: Supplementary file 1 — Table S1 [file FSN3-9-672-s001.docx]

Table S1 Nonlinear fitting parameters of different microbial growth kinetics equations of *L. plantarum* Z7

| Culture medium | Modified Gompertz equation | | | Modified Logistic equation | | | |
| --- | --- | --- | --- | --- | --- | --- | --- |
|  | μ_max_ (h^-1^) | λ(h) | R^2^ | A | μ_max_ (h^-1^) | λ(h) | R^2^ |
| MRS | 0.26342 | 2.66641 | 0.96705 | 1.57847 | 0.28377 | 2.96351 | 0.98231 |
| FOS | 0.28242 | 1.79796 | 0.99108 | 1.79634 | 0.2855 | 2.04706 | 0.9925 |
| 0.5%WOPs | 0.2957 | 1.45615 | 0.9985 | 2.18247 | 0.31308 | 1.85177 | 0.99109 |
| 1.0% WOPs | 0.35695 | 1.70075 | 0.99419 | 2.38674 | 0.37247 | 2.03321 | 0.99419 |
| 1.5% WOPs | 0.37138 | 1.50005 | 0.99663 | 2.96498 | 0.3908 | 1.81814 | 0.98691 |
| 2.0% WOPs | 0.6932 | 2.03145 | 0.99661 | 3.09466 | 0.69463 | 2.22165 | 0.99735 |
