# Supplementary material for: Beneficial effect and mechanism of walnut oligopeptide on Lactobacillus plantarum Z7
Source: Food Sci Nutr. 2021 Jan 8;9(2):672–81. doi: 10.1002/fsn3.2029 (PMC7866578; doi:10.1002/fsn3.2029)
Supplement: Supplementary file 2 — Table S2 [file FSN3-9-672-s002.docx]

Table S2 Assignment of Raman bands in biofilms

| Band or band range (cm^-1^) | Assigned to | Reference |
| --- | --- | --- |
| 544-553 | Carbohydrate | ([Wepf, Beese, & Krupinska, 2008](#_ENREF_8))  ([K. Christian Schuster, 2000](#_ENREF_3)) |
| ~600 | Arom ring def sporopollenin, phenylalanine |  |
| 720-730 | CH_2_, adenine; ring breath tryptophan |  |
| 810-820 | C-O-P-O-C  in RNA, nucleic acids |  |
| 820-860 | C-C str, C-O-C glycosidic link, C-O-P-O-C  in RNA |  |
| 855-899 | C-O-C 1,4 glycosidic link, C-C str | ([Wepf, Beese, & Krupinska, 2008](#_ENREF_8)) |
| 1001-1005 | Phenylalanine | ([K.C. Schuster, 2000](#_ENREF_4)) |
| 1030-1130 | Carbohydrates, mainly -C-C-, C-O, C-O-H def |  |
| 1145-1160 | C-C, C-O ring breath, asymm | ([Ivleva, Niessner, & Panne, 2005](#_ENREF_2)) |
| 1200-1290 | Amide III |  |
| ~1300 | N-H, C-H def, amide III |  |
| ~1320 | Protein (amide III); C-H def | ([Wagner, Ivleva, Haisch, Niessner, & Horn, 2009](#_ENREF_6)) |
| ~1327 | Protein (amide III); C-H def |  |
| 1343 | Protein (amide III) | ([Wei E. Huan, 2004](#_ENREF_7)) |
| 1400-1420 | COO^-^ symm |  |
| 1440-1460 | CH_2_ def | ([Rosch, Harz, Schmitt, Peschke, Ronneberger, Burkhardt, et al., 2005](#_ENREF_5)) |
| 1574 | Guanine, adenine  (ring stretching) |  |
| 1583 | Guanine, adenine ring str |  |
| ~1600 | Arom ring str, sporopollenin, phenylalanine | ([K.C. Schuster, 2000](#_ENREF_4)) |
| 1605-1620 | C=C Tyrosine, tryptophan, phenylalanine |  |
| 1540-1650 | COO^-^ asymm | ([Wagner, Ivleva, Haisch, Niessner, & Horn, 2009](#_ENREF_6)) |
| 1650–1680 | Amide I and unsaturated lipids |  |
| 1725-1750 | C=O str |  |
| 2920–1855 | CH_2_, CH_3_ str, sporopollenin, lipids | ([Harz, Rosch, Peschke, Ronneberger, Burkhardt, & Popp, 2005](#_ENREF_1)) |
| 2935 | CH_2_ str asymmetric |  |

str: stretching; def: deformation; arom: aromatic.

**References**

Harz, M., Rosch, P., Peschke, K. D., Ronneberger, O., Burkhardt, H., & Popp, J. (2005). Micro-Raman spectroscopic identification of bacterial cells of the genus Staphylococcus and dependence on their cultivation conditions. *Analyst, 130*(11), 1543-1550.

Ivleva, N. P., Niessner, R., & Panne, U. (2005). Characterization and discrimination of pollen by Raman microscopy. *Anal. Bioanal. Chem., 381*(1), 261-267.

Schuster, K. C., Reese, I., Urlaub, E., Gapes, J. R., Lendl, B. (2000). Multidimensional information on the chemical composition of single bacterial cells by confocal Raman microspectroscopy. *Anal. Chem., 72,* 5529-5534.

Schuster, K.C., Urlaub, E., Gapes, J. R. (2000). Single-cell analysis of bacteria by Raman microscopy: spectral information on the chemical composition of cells and on the heterogeneity in a culture. *J. Microbiol. Methods, 42*, 29-38.

Rosch, P., Harz, M., Schmitt, M., Peschke, K. D., Ronneberger, O., Burkhardt, H., Motzkus, H. W., Lankers, M., Hofer, S., Thiele, H., & Popp, J. (2005). Chemotaxonomic identification of single bacteria by micro-Raman spectroscopy: application to clean-room-relevant biological contaminations. *Appl. Environ. Microbiol., 71*(3), 1626-1637.

Wagner, M., Ivleva, N. P., Haisch, C., Niessner, R., & Horn, H. (2009). Combined use of confocal laser scanning microscopy (CLSM) and Raman microscopy (RM): Investigations on EPS – Matrix. *Water Res., 43*(1), 63-76.

Huang, W. E., Griffiths, R. T., Thompson, I. P., Bailey, M. J., Whiteley, A. S. (2004). Raman Microscopic Analysis of Single Microbial Cells. *Anal. Chem, 76*, 4452-4458.

Wepf, R., Beese, M., & Krupinska, K. (2008). Reevaluation of the documentation of the technique used for the work “Combined use of confocal laser scanning microscopy and transmission electron microscopy for visualization of identical cells processed by cryotechniques”. *Protoplasma, 232*(3-4), 267-269.
